# Supplementary material for: Comparative Study of Different H2S Donors as Vasodilators and Attenuators of Superoxide-Induced Endothelial Damage
Source: Antioxidants (Basel). 2023 Feb 1;12(2):344. doi: 10.3390/antiox12020344 (PMC9951978; doi:10.3390/antiox12020344)
Supplement: Supplementary file 1 [file antioxidants-12-00344-s001.zip › antioxidants-2189790-supplementary.pdf]

Supporting Information

Comparative study of different H2S donors as vasodilators and attenuators of superoxide-induced endothelial damage

Elisabetta Marini[1], Barbara Rolando[1], Federica Sodano[1,2], Federica Blua[1], Giulia Concina[3], Stefano Guglielmo[1], Loretta Lazzarato[1], and Konstantin Chegaev[1,*]

[1] Department of Drug Science and Technology, University of Turin, 10125 Turin, Italy;

[2] Department of Pharmacy, "Federico II" University of Naples, 80131 Naples, Italy;

[3] Rita Levi-Montalcini Department of Neuroscience, University of Turin, 10125 Turin, Italy

Corresponding author: Konstantin Chegaev, konstantin.chegaev@unito.it.

Table of contents

Figure S1. Effect of **8** on acetylcholine-induced vasorelaxation in endothelium-intact rat aortic rings. S2

Table S1. Stability of compounds in pH=7.4 buffered solution (PBS) in the absence and in the presence of an excess of L-cysteine (50x), and calculated lipophilicity (CLOGP, clogD$^{7.4}$). S3-S4

Experimental section. Synthetic procedures for the preparation of compounds. S5-S6

Supplementary references S7

Figure S1. Effect of **8** (100 µM) on acetylcholine-induced vasorelaxation in endothelium-intact rat aortic rings. Control: rings incubated with vehicle only (DMSO) (straight line, □); rings incubated with **8** (straight line, ○); rings incubated with 500 µM pyrogallol (straight line, ■); rings incubated with **8** and 500 µM pyrogallol (dotted line, ●).

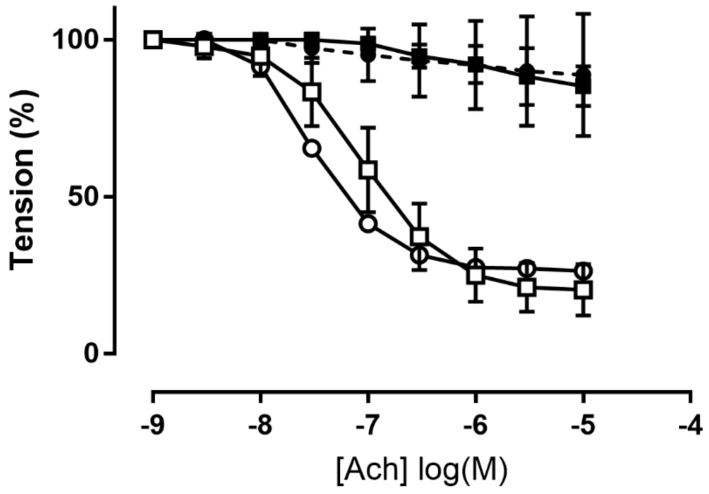

Table S1. Stability of compounds in pH=7.4 buffered solution (PBS) in the absence and in the presence of an excess of L-cysteine (50x), and calculated lipophilicity (CLOGP and clogD$^{7.4}$).

Compd	Structural formula	Stability (% compound ± SE)		Lipophilicity[1]	
		**Buffer** (pH =7.4; 37°C)	**Buffer** (pH =7.4; 37°C) **+ cysteine** (50×)	**CLOGP, clogD$^{7.4}$**	
1	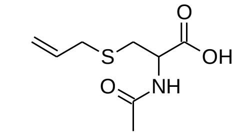	91.5 ± 1.0% 24h	87.3 ± 1.0% 1h<br>72.5 ± 1.0% 6h<br>69.5 ± 1.0% 24h	1.17	-1.83
**2**	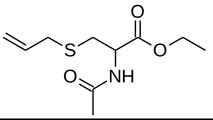	nd	nd	1.75	-
3	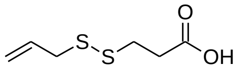	101.0 ± 1.0% 24h	$t_{1/2}$ ≈ 10 min	2.03	-0.97
4	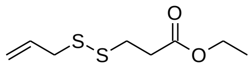	nd	nd	2.93	-
**5**	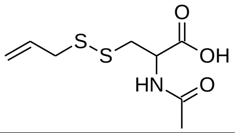	87.7 ± 1.0% 24h	$t_{1/2}$ ≈ 5 min	1.16	-1.84
**6**	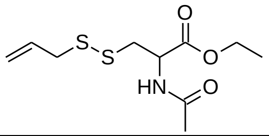	nd	nd	1.86	-
7	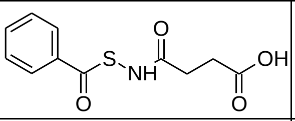	91.6 ± 1.1% 6h<br>57.1 ± 3.3% 24h	$t_{1/2}$ ≈ 1.3 h	1.43	-1.57
**8**	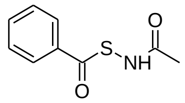	nd	nd	1.82	-
9	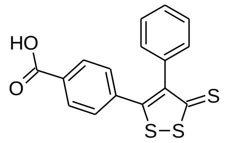	92.6 ± 0.8% 24h	98.6 ± 0.3% 1h<br>94.9 ± 0.7% 6h<br>78.1 ± 0.3% 24h	4.38	1.38
10	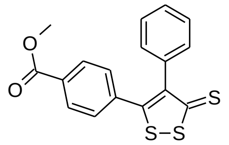	nd	nd	4.61	-
11	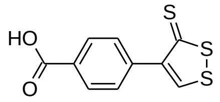	94.8 ± 3.2% 24h	99.0 ± 0.3% 1h<br>82.6 ± 0.2% 6h<br>37.6 ± 0.1% 24h	2.88	-0.12
12	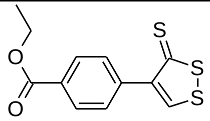	nd	nd	3.64	-
13	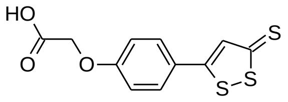	101.8 ± 1.4% 24h	99.0 ± 0.4% 1h<br>96.6 ± 0.1% 6h<br>81.9 ± 0.1% 24h	2.91	-0.09

14	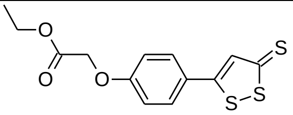	nd	nd	3.77	-
15	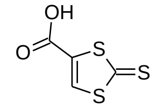	100.0 ± 1.0% 24h	100.0 ± 0.2% 1h<br>96.6 ± 1.0% 24h	1.90	-1.1
16	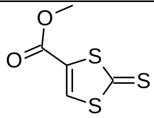	nd	nd	2.12	-
17	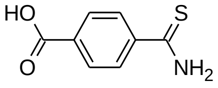	82.3 ± 0.4% 24h	97.6 ± 0.2% 1h<br>84.1 ± 0.2% 6h<br>25.0 ± 0.6% 24h	1.53	-1.47
18	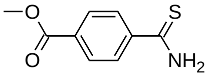	nd	nd	1.84	-

[1] Partition coefficients of compounds in neutral form (CLOGP) and ionized form ($clogD^{7.4}$) were calculated using Bio-Loom for Windows v.1.5 (BioByte Corp. Claremont, CA, USA).

Experimental Section

Chemistry. ^{1}H and ^{13}C NMR spectra were recorded on a BrukerAvance 300, at 300 and 75 MHz, respectively, using solvent residual peak as internal standard. The following abbreviations indicate peak multiplicity: *s* = singlet, *d* = doublet, *t* = triplet, *m* = multiplet, *br.* = broad. ESI MS spectra were recorded on a Micromass Quattro API micro (Waters Corporation, Milford, MA, USA) mass spectrometer. Data were processed using a MassLynxSystem (Waters). Flash column chromatography was performed on silica gel (Merck Kieselgel 60, 230–400 mesh ASTM). The progress of the reactions was followed by thin-layer chromatography (TLC) on 5 × 20 cm plates Merck Kieselgel 60 F_{254}, with a layer thickness of 0.20 mm. Anhydrous sodium sulfate (Na_2SO_4) was used as drying agent for the organic phases. Organic solvents were removed under reduced pressure at 30 °C. Synthetic-purity solvents acetone, dichloromethane (DCM), hexane, ethyl acetate (EtOAc), diisopropyl ether (*i*-Pr_2O), dimethylformamide (DMF) and 40–60 petroleum ether (PE) were used. Dry DMF was obtained through storage on 4Å molecular sieves. Commercial starting materials were purchased from Sigma-Aldrich, Fluorochem, and TCI Europe. Compound **1** [1], **3** [2], **4** [1], **5** [3], **8** [4], **9** [1], **10** [1], **11** [2], **13** [1], **14** [1], **15** [5], **16** [6], **17** [1], **18** [1] were synthetized as described elsewhere.

General synthetic procedure for the synthesis of ethyl esters (**2**, **6**, **12**)
To the solution of appropriate carboxylic acid (1.0 mmol) in dry DMF (10 mL) KF (175 mg, 3.0 mmol) was added followed by the addition of EtI (0.24 mL, 3.0 mmol) and the reaction was stirred at rt until completed (TLC control). Solvent was evaporated under reduced pressure and obtained oily residue was dissolved in H_2O / DCM (25 mL / 25 mL) mixture. Organic phase was separated, washed with $NaHCO_3$ sat. sol. (20 mL), brine and dried. Solvent was evaporated and products were isolated as described.

Ethyl (acetylamino)(prop-2-en-1-ylsulfanyl)acetate (**2**). Obtained oil was purified by flash chromatography (eluent PE / EtOAc 7 / 3 v / v) and then crystallized from hexane to give a white powder. Yield: 80 mg; 35 %. ^{1}H-NMR ($CDCl_3$) δ: 1.31 (*t*, 3H, J^3_{HH} = 7.2 Hz, CH_2CH_3), 2.06 (*s*, 3H, $COCH_3$), 2.89 (*dd*, 1H, J^3_{HH} = 5.3 Hz, J^2_{HH} = 13.8 Hz) and 2.97 (*dd*, 1H, J^3_{HH} = 4.8 Hz, J^2_{HH} = 13.8 Hz) ($OOCCHCH_2$), 3.13 (*d*, 2H, J^3_{HH} = 7.1 Hz, $CH_2CH=CH_2$), 4.24 (*q*, 2H, J^3_{HH} = 7.2 Hz, CH_2CH_3), 4.67 – 4.83 (*m*, 1H, $CH=CH_2$), 5.10 – 5.15 (*m*, 2H, $CH=CH_2$), 5.68 – 5.82 (*m*, 1H, OOCC*H*), 6.27 (*br.d.*, 1H, N*H*). MS (ESI$^+$) *m/z* 232.0 $(M+H)^+$.

Ethyl (acetylamino)(prop-2-en-1-yldisulfanyl)acetate (**6**). Obtained oil was purified by flash chromatography (eluent PE / EtOAc 7 / 3 v / v) and then crystallized from hexane to give a white powder. Yield: 75 mg (29 %). ^{1}H-NMR ($CDCl_3$) δ: 1.29 (*t*, 3H, J^3_{HH} = 7.1 Hz, CH_2CH_3), 2.04 (*s*, 3H, $COCH_3$), 3.10 – 3.24 (*m*, 2H, $OOCCHCH_2$), 3.32 (*d*, 2H, J^3_{HH} = 7.7 Hz, $CH_2CH=CH_2$), 4.22 (*q*, 2H, J^3_{HH} = 7.1 Hz, CH_2CH_3), 4.82 – 4.88 (*m*, 1H, $CH=CH_2$), 5.13 – 5.22 (*m*, 2H, $CH=CH_2$), 5.75 – 5.89 (*m*, 1H, OOCC*H*), 6.44 (*br.d.*, 1H, N*H*); ^{13}C-NMR ($CDCl_3$) δ: 14.0, 23.1, 40.6, 42.1, 51.9, 61.9, 118.9, 133.0, 169.8, 170.5. MS (ESI$^+$) *m/z* 286.3 $(M+Na)^+$.

Ethyl 4-(3-thioxo-3H-1,2-dithiol-4-yl)benzoate (**12**). Obtained oil was purified by flash chromatography (eluent PE / acetone 9 / 1 v / v) and then crystallized from *i*-Pr_2O to give a orange powder. Yield: 85 mg (30 %).^{1}H-NMR ($CDCl_3$) δ: 1.41 (*t*, 3H, J^3_{HH} = 7.0 Hz, CH_2CH_3), 4.41 (*q*, 2H, J^3_{HH} = 7.0 Hz, CH_2CH_3), 7.63 (*m*, 2H), 8.13 (*m*, 2H) (C_6H_4), 8.48 (*s*, 1H, C*H*); ^{13}C-NMR ($CDCl_3$) δ: 14.3, 61.1, 128.9, 129.6, 130.8, 137.4, 148.2, 154.8, 166.0. MS (ESI$^+$) *m/z* 283.2 $(M+H)^+$.

Synthesis of 4-[(benzoylsulfanyl)amino]-4-oxobutanoic acid (**7**). To the solution of thiobenzoic acid (2.0 mL, 17.0 mmol) in 2M sol of KOH (17.0 mL, 34.0 mmol) placed in the ice bath and under positive N_2 pressure hydroxylamine-*O*-sulfonic acid (1.95 g, 17.2 mmol) was added. Ice bath was removed and reaction was allowed to reach rt. Abundant white precipitate was filtered off, washed with small portions of ice-cold water and desiccated. Obtained solid was dissolved in DCM (25 mL) and succinic anhydride (3.4 g, 34.0 mmol) was added. Reaction was stirred at rt overnight. Obtained white precipitated was filtered off, washed with small portion of cold DCM and crystallized from hot *i*-Pr_2O to give a title compound. Yield: 1.25g (29%). ^{1}H-NMR (DMSO-d_6) δ: 2.54 (*t* overlapped with solvent signal, 2H, J^3_{HH} = 6.5 Hz, CH_2CH_2), 2.67 (*t*, 2H, CH_2CH_2), 7.60 (*m*, 2H), 7.75 (*m*, 1H), 7.88 (*m*, 2H) (C_6H_5), 9.83 (*s*, 1H, N*H*), 12.22 (*br.s.*, 1H, COOH); ^{13}C-NMR (DMSO-d_6) δ: 29.0, 30.5, 126.6, 129.5, 133.9, 134.6, 173.2, 173.5, 190.8. MS (ESI$^-$) *m/z* 252.0 (M-H)$^-$.

1. Chegaev, K.; Rolando, B.; Cortese, D.; Gazzano, E.; Buondonno, I.; Lazzarato, L.; Fanelli, M.; Hattinger, C.M.; Serra, M.; Riganti, C.; Fruttero, R.; Ghigo, D.; and Gasco, A. H_2S-Donating doxorubicins may overcome cardiotoxicity and multidrug resistance. *J. Med. Chem.* **2016**, *59*, 4881−4889.
2. Lee, M.; Tazzari, V.; Giustarini, D.; Rossi, R.; Sparatore, A.; Del Soldato, P.; McGeer, E.; McGeer, P.L. Effects of hydrogen sulfide-releasing L-DOPA derivatives on glial activation. *J. Biol. Chem.* **2010**, *23*, 17318-17328.
3. Izigov, N.; Farzam, N.; Savion, N. S-allylmercapto-N-acetylcysteine up-regulates cellular glutathione and protects vascular endothelial cells from oxidative stress. *Free Radic Biol Med.* **2011**, *50(9)*, 1131-9.
4. Zhao, Y.; Yang, C.; Organ, C.; Li, Z.; Bhushan, S.; Otsuka, H.; Pacheco, A.; Kang, J.; Aguilar, H.C.; Lefer, D.J.; Xian, M. Design, Synthesis, and Cardioprotective Effects of N-Mercapto-Based Hydrogen Sulfide Donors. *J. Med. Chem.* **2015**, *58*, 7501−7511.
5. Dartigues, B.; Cambar, J.; Trebaul, C.; Brelivet J.; Guglielmetti, R. Proprietes diuretiques de derives des dithiole-thiones. Recherche d'une relation structure-activite. *Eur. J. Med. Chem.* **1980**, *15*, 405-412.
6. Terauchi, T.; Kobayashi, Y.; Misaki, Y. Synthesis of bis-fused tetrathiafulvalene with mono- and dicarboxylic acids. *Tetrahedron Letters,* **2012**, *53*, 3277–3280.
